# Influence of Post-Bond Heat Treatment on Microstructure and Creep Behavior of the Brazed Single-Crystal Nickel Superalloy

**DOI:** 10.3390/ma15124053

**Published:** 2022-06-07

**Authors:** Xingyu Hou, Shiyang Wang, Keqiang Qiu, Yuan Sun, Yanhong Yang, Yizhou Zhou

**Affiliations:** 1 School of Materials Science and Engineering, Shenyang University of Technology, Shenyang 110023, China; xyhou@imr.ac.cn; 2 Institute of Metal Research, Chinese Academy of Sciences, Shenyang 110016, China; yhyang@imr.ac.cn (Y.Y.); yzzhouimr@163.com (Y.Z.)

**Keywords:** single-crystal Ni superalloy, brazing, post-bond heat treatment (PBHT), microstructure, creep life, diffusion affected zone

## Abstract

Post-bond heat treatment (PBHT) is an effective way to improve the bonding quality of a brazed joint. Herein, brazing of a nickel-based single crystal superalloy is carried out using a Ni-Cr-Co-B-Si-Al-Ti-W-Mo filler alloy, and the microstructure and creep property of the brazed joint are systematically investigated using scanning electron microscopy (SEM), Thermo-Calc software, an electron probe micro-analyzer (EPMA), X-ray diffractometer, confocal scanning laser microscope (CSLM), and transmission electron microscopy (TEM). The results reveal that the as-prepared joint only consists of an isothermally solidified zone (ISZ) and an athermally solidified zone (ASZ), where the cubic γ′ phase is observed in the ISZ, and skeleton-like M_3_B_2_, γ + γ′ eutectic and reticular G phases are observed in the ASZ. Furthermore, the γ + γ′ eutectic and G phases disappear and the M_3_B_2_ alters from a skeleton-like to block-like shape in the ASZ after PBHT. Meanwhile, some lath-like M_3_B_2_ phases are precipitated at the edge of the ISZ and several M_3_B_2_ phases are precipitated in the base metal, forming a new zone in the brazed joint, namely at the diffusion affected zone (DAZ). Owing to the removal of low melting point eutectics from the as-prepared joint, the creep life also increases from 188 h to 243 h after PBHT. The current work provides a method for the optimization of brazed joints based on the Ni-based single crystal superalloy.

## 1. Introduction

Nickel-based single crystal superalloys are promising materials for hollow turbine blades and other components of the hot end of aero-engines with a high thrust-to-weight ratio due to their excellent high-temperature mechanical properties and high resistance to oxidation and corrosion [1,2,3]. Owing to the addition of several refractory elements such as W, Mo, Ta, Nb, and Re in the alloys and the increasingly intricated design of the internal cooling channels, manufacturing these components solely by casting techniques is a challenging task [4,5,6,7]. Therefore, it is necessary to adopt a suitable joining technology to improve the product qualification rate and material utilization rate of the parts with a complex hollow structure [8,9,10,11].

Furthermore, several γ′ forming elements, such as Al, Ti, Ta, and Hf, are added to elevate the high-temperature strength of the superalloy; however, the addition of these elements leads to poor weldability during fusion welding [12,13,14,15]. For thin-walled turbine blades with complex structures, fusion welding can easily change the dimensional accuracy of the blades and the microstructure of matrix materials, and may even lead to hot cracks, thus reducing the mechanical properties of blade materials [16,17]. Nevertheless, brazing can fundamentally solve this problem by heating joint uniformly. The addition of a melting point depressant (MPD) such as B and Si improves the solder fluidity and reduces the joining temperature to avoid cracks and achieve high-performance joints [18,19,20]. Although a solder with a lower melting point reduces the temperature-bearing capacity of the joint to a certain extent, the optimization of the blade bonding structure and the application of a thermal barrier coating material help brazing joints have certain advantages in terms of size, crack, and microstructure control over other bonding methods in the joining of turbine blade materials [21].

However, the addition of MPD often leads to the emergence of hard and brittle compounds in the joint [22,23,24]. Consequently, PBHT is utilized to transform or decompose the brittle compounds and to homogenize the microstructure of the joint. For instance, Wang et al. [25] have observed that the decomposition of boride and other eutectics in the brazing joint of a nickel-based single crystal superalloy during PBHT promotes microstructural homogenization and formation of grain boundaries. Xu et al. [26] have carried out brazing on CMSX-4 alloy and demonstrated that the elemental distribution became uniform, brittle compounds were partially dissolved, and brittle compounds were partially embedded in the single crystal joint after heat treatment. Hinchy et al. [27] have analyzed the effect of multiple thermal cycles on the microstructural transformation of the brazed joint of a single crystal nickel-based superalloy and indicated that multiple thermal cycles could lead to a uniform brazing joint. According to the literature [25,26,27], the joint evolution during PBHT can be commonly described as microstructural homogenization and phase transformation, while the re-growth of the isothermal solidification layer and formation of new regions rarely occur. However, the evolution of different areas in a joint should be studied to optimize the PBHT.

Joints with excellent microstructural uniformity after PBHT usually render superior mechanical properties [28,29,30]. Amiri et al. [28] have investigated the microstructure and shear strength of the joints of GTD-111 superalloy with dissimilar heat treatment cycles and obtained optimal performance by using the following joining sequence: joining → full-solution → partial solution → aging heat treatment. Wang et al. [29] have studied the evolution of γ′ phases in the joints of a γ′-strengthened Co-based single crystal superalloy during PBHT and demonstrated that the volume fraction and size of γ′ phases increased after PBHT, improving the microhardness of joint. Liu et al. [30] have considered that the existence of hard and brittle borides in Mar-M247 joints reduced the mechanical properties and suggested these borides should be eliminated to obtain an optimal mechanical performance.

One should note that the PBHT is a promising route to remove the brittle phases and improve the mechanical properties. Nevertheless, previous studies were mainly focused on the shear strength [28,31,32,33,34] and a few studies focused on the effect of microstructural evolution and creep behavior of the joint. The creep life is an important performance index because the failure of components is usually induced by long-lasting stress.

Therefore, the current study mainly focuses on the microstructural evolution of the joint, which is prepared by using a single crystal nickel-based superalloy and brazed by a novel filler metal containing Al and Ti elements. Moreover, PBHT is carried out to eliminate the brittle borides. The microstructural evolution of different joint regions during BPHT, including phase dissolution, phase formation, and zone formation, is studied in detail, and the influence of PBHT on high-temperature creep behavior is also demonstrated.

## 2. Experimental Procedure

Herein, a solid-solution heat-treated nickel-based single crystal superalloy (named as Ni-Al-Co-Cr-Ta-W-Re-Hf-Mo), directionally solidified with an orientation of [001], was used as the base material (BM), and a Ni-Cr-Co-B-Si-Al-Ti-W-Mo powder was used as the filler metal. The chemical composition of both base material and filler is given in Table 1. The plane to be bonded was perpendicular to the [001] orientation of the BM and the width of the preset brazing seam was approximately 50 μm. The brazing experiments were carried out using a vacuum brazing furnace at 1230 °C for 30 min, where the vacuum was not worse than 4.0 × 10^−2^ Pa. The post-bond heat treatment consisted of heating at 1150 °C for 4 h, followed by air cooling, and heating at 870 °C for 24 h, followed by air cooling.

**Figure 1:**
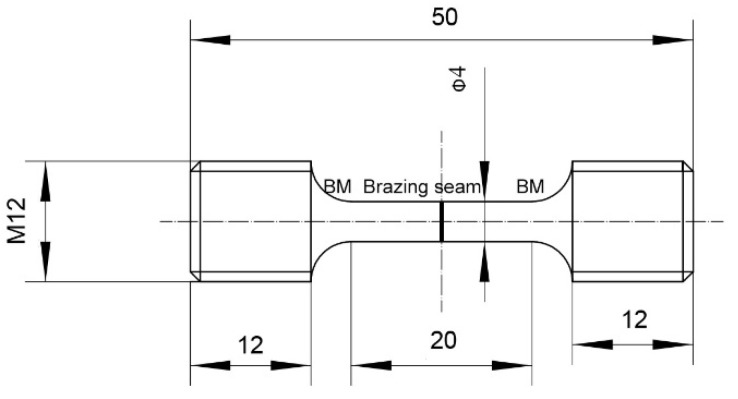
Drawing of a specimen for creep testing.

The creep tests were conducted at 980 °C/100 MPa using a creep testing machine (NCS GNCJ-50, Beijing, China). Figure 1 shows the drawing of a specimen for creep testing. The loading direction was aligned with the [001] direction of the BM, and the load was set as 1257 N. FEI Inspect F50, coupled with an X-ray energy dispersive spectroscopy (EDS), was utilized for SEM observations and EDS line scans of the joint samples. It was also utilized for creep behavior analysis of the creep testing samples and semi-quantitative compositional analysis of each phase on the fracture surfaces after creep tests. The atomic composition of phases in the joint samples was quantitatively analyzed by EPMA (Shimadzu Model 1610, Kyoto, Japan). Theoretical formation temperatures and volume fractions of different phases in the as-prepared joint were calculated using Thermo-Calc software with TTNi8 database (Version 8, Thermo-Calc Software AB, Stockholm, Sweden) according to the chemical composition of the filler metal. The phase composition was characterized using a X-ray diffractometer (Bruker D8 DISCOVER A25, Karlsruhe, Germany) with Cu-Kα radiation and a TEM (FEI Tecnai G2 20, Hillsboro, OR, USA). The microstructural evolution of the ASZ during the heating process was observed in situ using a CSLM (OLYMPUS VL2000DX-SVF17SP, Tokyo, Japan). The surfaces of specimens for X-ray diffraction and CSLM observation were ground with 1500 rit SiC sandpaper and ultrasonically cleaned in acetone for 20 min.

## 3. Results and Discussion

### 3.1. Microstructure of the As-Prepared Joint

**Figure 2:**
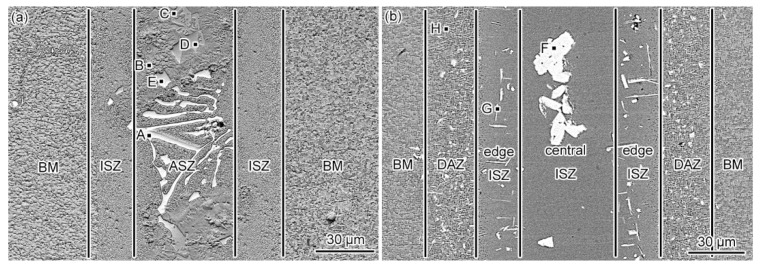
The microstructure of (**a**) as-prepared and (**b**) PBHT-processed joints.

**Figure 3:**
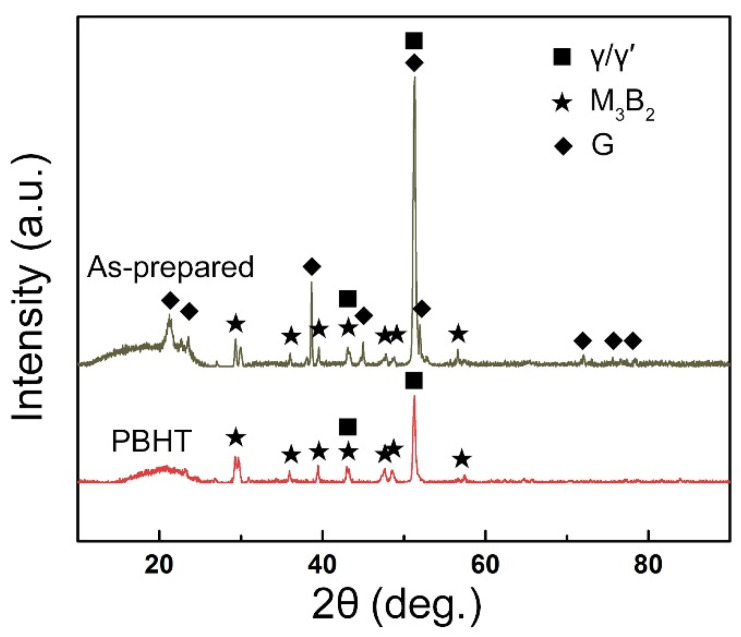
XRD patterns of the as-prepared and PBHT-processed joints.

Figure 2a shows the typical microstructure of the as-prepared joint. Based on the morphology, the as-prepared joint consisted of two different zones: isothermally solidified zone (ISZ) and athermally solidified zone (ASZ). The ASZ lies at the center of the joint, whereas the ISZ lies adjacent to the base metal (BM). Different from the previously investigated bonding joints [15,18,25,26,27], each of which contains a diffusion affected zone (DAZ), we have not observed any precipitates near the BM. Hence, DAZ is not formed in the as-prepared joint. X-ray diffraction (XRD) patterns of the as-prepared joint are presented in Figure 3 (black line), confirming the presence of γ/γ′, M_3_B_2,_ and G phases.

**Figure 4:**
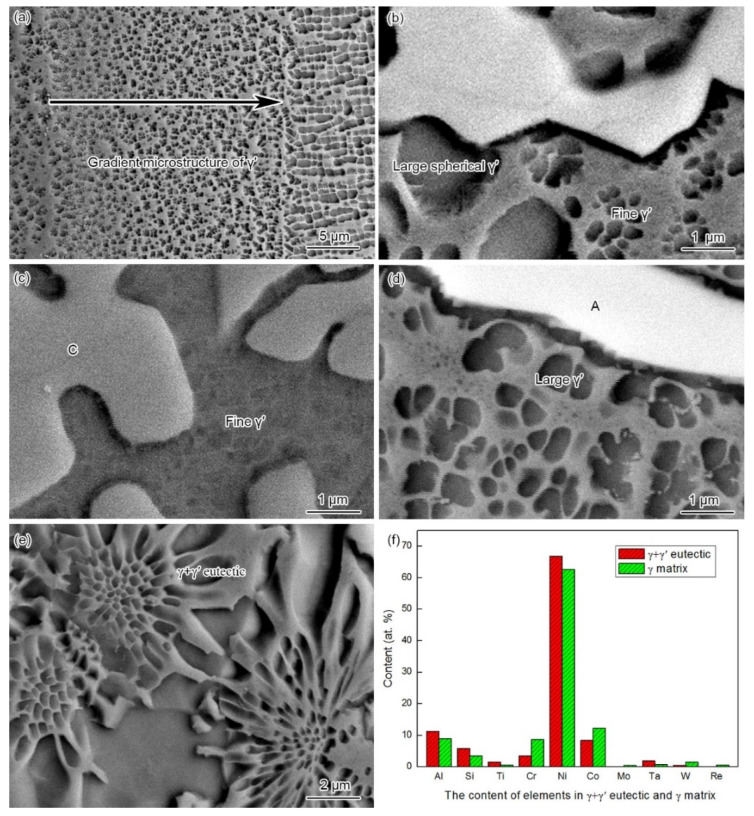
Microstructure of brazing joints: (**a**) gradient microstructure of γ′ precipitates in the ISZ; (**b**) large spherical eutectic γ′ and fine γ′ precipitates in the ASZ; (**c**) fine γ′ precipitates near Area-C; (**d**) large γ′ precipitates around Area-A (**e**) γ + γ′ eutectic (Area-B); and (**f**) chemical composition of γ + γ′ eutectic (Area-B) and γ matrix.

**Figure 5:**
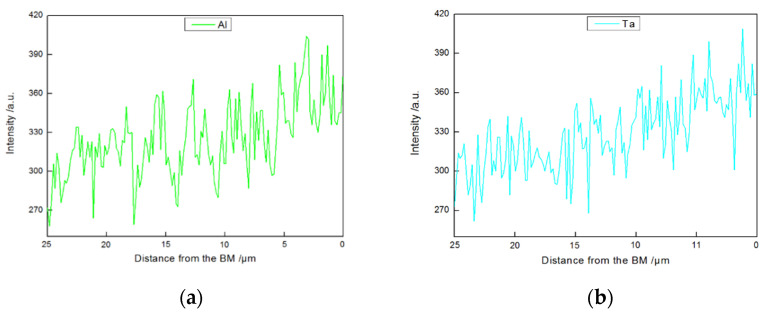
The EDS line scans of Al and Ta elements in the ISZ of the brazing joints, where the scan direction corresponds to the arrow in Figure 4a: (**a**) Al element (**b**) Ta element.

The ISZ was mainly composed of γ matrix and γ′ phase (Figure 2a and Figure 5a). The width of the ISZ was 25 μm, which accounted for around 25% of the total width of the joint. The size and volume fraction of the ISZ-γ′ phase exhibited a gradient distribution, increasing from the ASZ margin to BM (Figure 4a). Furthermore, we have analyzed the chemical composition to understand the formation mechanism of the gradient distributed ISZ-γ′ phase. The contents of Al and Ta in the ISZ are higher than the filler metal (see ISZ in Table 1 and Table 2), increasing from the ASZ to BM (Figure 5). The higher content of Al and Ta in the ISZ should account for the dissolution of BM during the bonding process, resulting in the transfer of BM elements to the liquid interlayer.

This process can be explained as follows: at the outset of the joining process, the interactions between the molten filler metal and adjacent BM changed the composition of BM at the solid/liquid interface. Then, the fusion temperature of the BM at the interface dropped, resulting in the dissolution of the BM into the vicinal liquid filler metal. Herein, the solid/liquid interface moved towards the BM and the melting point depressants (MPD) elements (B and Si) diffused into the BM, increasing the melting point of the liquid. Once the melting point of the liquid filler metal increases to the bonding temperature (1230 °C), the solid/liquid boundary reaches a transient state of equilibrium [31,35,36]. The content of Ta, Re, W, and Al in the BM is higher than the original filler metal, which continues to increase in the liquid state. With the extension of holding time, the interdiffusion of elements leads to an increase in the melting point of liquid at the interface. Hence, the liquid/solid interface starts to move in the reverse direction towards the liquid side. At this stage, B and Si continuously diffuse across the freshly-formed γ-Ni layer to the depth of BM, and the isothermal solidification layer constantly grows towards the liquid phase [35,37,38,39]. During isothermal solidification, γ′ forming elements such as Al and Ta from the BM diffuse along the newly formed γ-Ni to the center of liquid phase, resulting in a gradient distribution of these elements in the ISZ (Figure 5a). Additionally, the precipitates in the BM cannot be observed in the as-prepared joint due to the insufficient holding time. Furthermore, the diffused MPDs do not exceed the solubility of BM.

**Figure 6:**
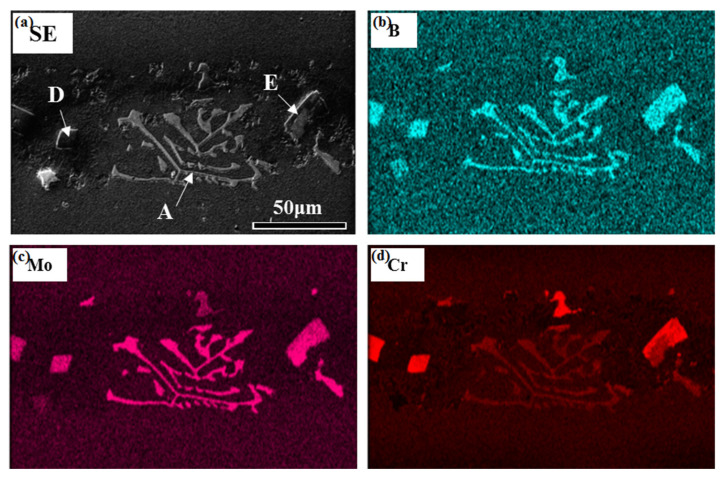
The elemental distribution maps of the as-prepared joint (Area-A, Area-D, and Area-E in the SE image represent skeleton-like white phase, block-like gray phase, and block-like gradient phase, respectively) (**a**) SE image (**b**) B element distribution map (**c**) Mo element distribution map (**d**) Cr element distribution map.

**Figure 7:**
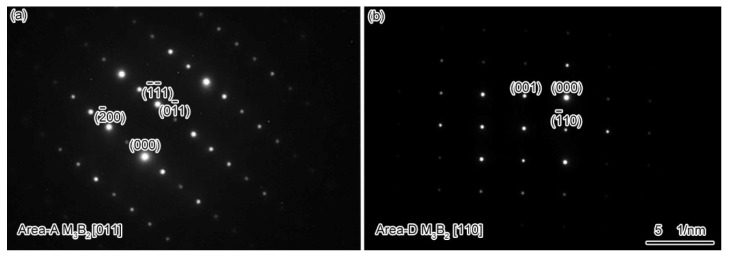
Identification of phases in the brazing joint: (**a**) Area-A M_3_B_2_ (**b**) Area-D M_3_B_2_.

The microstructure of the ASZ is quite complex (Figure 2a). Several precipitates can be observed based on the image contrast, which can be classified as large skeleton-like white phase (Area-A), flower-like eutectics (Area-B), reticular gray phase (Area-C), block-like gray phase (Area-D), and block-like gradient phase (Area-E). As shown in Table 2, Area-A, -D, and -E contain almost the same content of B (~40 at.%) with different contents of W, Mo, and Cr, where Area-A contains a higher amount of W and Mo while Area-D contains a higher amount of Cr (Figure 6). The atomic ratios of B in these precipitates are closer to the M_3_B_2_ phase, as evidenced by the XRD analysis (Figure 3) and the TEM analysis (Figure 7). Hence, the phase in Area-A, -D, and -E can be termed as M_3_B_2_. According to the morphology and chemical composition of Area-B, it can be concluded that Area-B is γ + γ′ eutectic phase (see Figure 4e,f). The content of Ni in Area-C is 54 at.% and it is rich in Si and Ti, corresponding to the composition of G phase (Ni_16_Ti_6_Si_7_, Figure 3).

**Figure 8:**
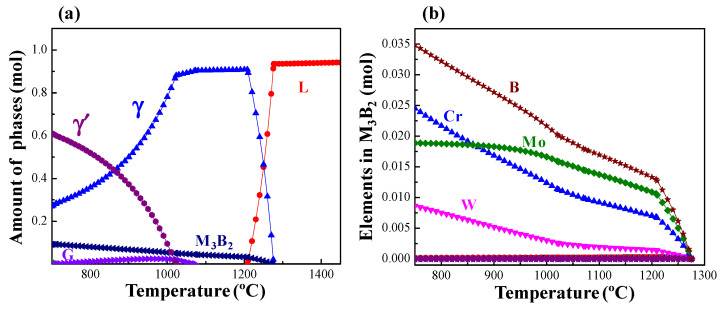
Thermo-Calc predictions for the ASZ of brazing joints based on the TTNi8 database: (**a**) Contents of phases at different temperatures (**b**) Contents of elements in M_3_B_2_ phase at different temperatures.

The solidification of the ASZ should be studied to understand the formation mechanism of the precipitates in the ASZ. Figure 8a illustrates the Thermo-Calc simulated results of the solidification process. According to the theoretical results, the M_3_B_2_ phase is the first to form during the cooling process, followed by the G phase. Finally, the γ′ phase precipitates in the γ matrix. However, it is known that the simulated results are based on the equilibrium solidification process and some nonequilibrium solidification process cannot be predicted. Therefore, the actual solidification should be more complex. Even so, the calculated result can qualitatively describe the formation sequence of the basic phases during the cooling process. As shown in Figure 2a, the γ + γ′ eutectic prefers to locate at the ISZ/ASZ interface, hence, the γ + γ′ eutectic is more likely to form first during the cooling process. As the γ + γ′ eutectic forms, B and Si are repelled to the residual liquid. The EPMA results (Table 2) indicate the skeleton-like white M_3_B_2_ (Area-A in Figure 2a) mainly contains Cr, W, Mo, and B. Therefore, B combines with Cr, W, and Mo atoms to form the primary M_3_B_2_ phase (skeleton-like white M_3_B_2_). Simultaneously, the primary M_3_B_2_ phase continually nucleates and grows within the liquid phase. Owing to the consumption of B element, the liquidus of the liquid phase around the M_3_B_2_ phase increased and the γ phase was formed beside the M_3_B_2_.

One should note that the D and E phases possess the same crystal structure as the A phase, namely the M_3_B_2_ phase. We have also observed the following phenomenon: the composition of A phase is similar to the light area of E phase (Table 2), and the composition of D phase is similar to the dark area of E phase. According to Figure 8b, the content of Cr in M_3_B_2_ increases faster than Mo with the decrease of temperature. Therefore, the pre-formed M_3_B_2_ should be higher in Mo and lower in Cr, and the latter-formed M_3_B_2_ should be the opposite. Thus, the formation sequence of the M_3_B_2_ phase can be given as: skeleton-like white M_3_B_2_ (Area-A), block-like gradient M_3_B_2_ (E phase), and block-like gray M_3_B_2_ (Area-D). As the formation and growth of the M_3_B_2_ phase consumed a large number of Cr, Mo, and W elements in the liquid phase, the contents of Si and Ti in the residual liquid phase increased, which promoted the formation of G phase. With the decrease of temperature, γ′ phase precipitates within the primary γ matrix. According to Ref. [38], during the solidification of a superalloy, the number and size of circular γ′ phase may increase with the decrease of the solidification rate or extension of the solidification temperature range. Owing to the furnace cooling in this experiment, the low cooling rate during solidification and a large temperature range resulted in the growth of γ′ phase into a large spherical shape. The γ′-forming elements (Al and Ti) in the nearby matrix entered these large spherical γ′ phases through long-range diffusion, which resulted in the decrease of the content of γ′-forming elements in the matrix, and the formation of fine γ′ phases in the nearby matrix (Figure 4b). Additionally, the size of γ′ around G phase (Figure 4c) is smaller than the M_3_B_2_ (Figure 4d) because a large number of γ′-forming elements are discharged from the M_3_B_2_ and promote the growth of γ′ phase, whereas the growth of G phase consumes the Ti element. Therefore, the size of γ′ phase precipitated from the surrounding area of phase C is distinctly smaller than that of phase A during the subsequent cooling process.

### 3.2. Microstructural Evolution of the PBHT-Processed Joint

**Figure 9:**
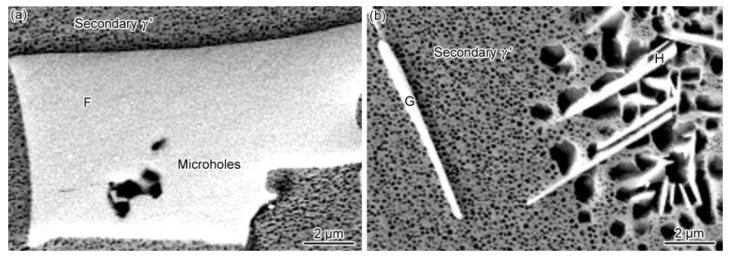
The microstructure of brazing joints after PBHT at 1150 °C/4 h (air cooling) and 870 °C/24 h (air cooling): (**a**) micro-holes in F phase and secondary γ′ precipitates in the central ISZ and (**b**) secondary γ′ precipitates and G phase at the edge of the ISZ and H phase in the DAZ.

**Figure 10:**
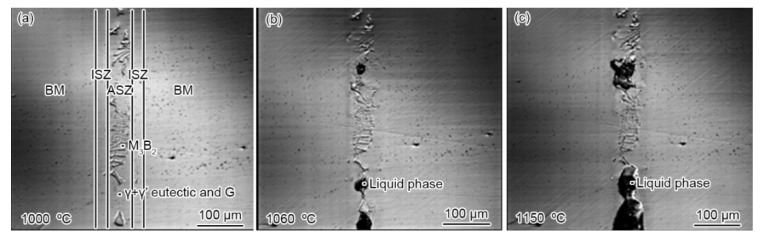
The microstructural evolution of the as-prepared joint during the heating process: microstructure at (**a**) 1000 °C (**b**) 1060 °C (**c**) 1150 °C.

Figure 2b presents the microstructure of the PBHT-processed joint. Only M_3_B_2_ and γ/γ′ phases were observed in SEM images, which is consistent with XRD results (Figure 3). The elimination of γ + γ′ eutectic and the reduction of the γ′ precipitates content in the PBHT-processed joint (Figure 2b) made the γ/γ′ line intensity decrease. Additionally, the content of borides in the center of the joint decreased after the PBHT (Figure 2b). The width of the ISZ in the PBHT-processed joint is significantly increased. Besides, several precipitates (Area-G and Area-H in Figure 2b) are observed in the ISZ and BM. Figure 9 shows the enlarged image of the PBHT-processed joint. Some aggregated block-like compounds (Area-F) can be observed in the ASZ, whereas the γ + γ′ eutectic and G phases disappear after PBHT. The content of Area-F is similar to the A phase (M_3_B_2_) in the as-prepared joint. According to the XRD results (Figure 3), F, G, and H phases belong to the M_3_B_2_-type borides. Moreover, a confocal scanning laser microscope (CSLM) was used to study the microstructural evolution of the joint during PBHT, as shown in Figure 10. At 1000 °C, there was no change in each area of the joint, indicating that each phase in the joint had not melted at this time. The joint started to melt in the ASZ at 1060 °C (the melted part of the joint appears black) and the γ + γ′ eutectic and G phases were liquefied when the temperature reached the PBHT temperature (1150 °C).

**Figure 11:**
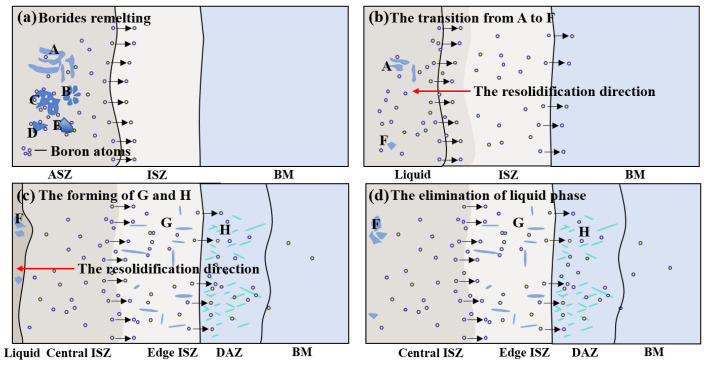
The schematic illustration of the microstructure evolution of the brazing joints during PBHT (A, B, C, D, E, F, G, H represent Area-A-B-C-D-E-F-G, and -H, respectively): (**a**) Borides remelting (**b**) The transition from A to F (**c**) The forming of G and H (**d**) The elimination of liquid phase.

Based on the above results, the microstructural evolution of the joint can be understood using the schematic in Figure 11. The high-temperature PBHT leads to the remelting of low-melting-point phases, such as γ + γ′ eutectic, G phase, and part of the borides in the ASZ. Additionally, the melting point depressants in the molten pool can diffuse to the BM during the heat preservation process (Figure 11a). This process eliminates most of the low-melting-point phases in the ASZ. As M_3_B_2_ is a high-temperature stable phase, it remains in the ASZ and transforms to the block-shaped phase during the heat preservation process (Figure 11b). During PBHT, several B and Si atoms are released due to the remelting of borides and silicide in the ASZ, entering the ISZ and BM regions. Nonetheless, the solubility of B atoms in the γ matrix at 1150 °C is much lower than that at 1230 °C (brazing temperature). Therefore, the supersaturated B reacts with strong boride forming elements, such as W, Mo, and Cr, to form lath-like M_3_B_2_ borides in the ISZ and the BM, which continue to grow during the holding process (Figure 11c,d).

It can be seen from Figure 2b that there is a fuzzy transition interface in the ISZ (edge ISZ/central ISZ in Figure 2b), and the contrast of the central ISZ is slightly darker than the edge ISZ in the BSE image. Therefore, it can be deduced that the edge ISZ is transformed from the original ISZ of the as-prepared joint, while the central ISZ is transformed from the ASZ. Their specific formation can be described as follows: the low-melting-point phases are remelted during BPHT and the isothermal solidification is restarted in the molten pool along the boundary of original ISZ to form the new central ISZ of the PBHT joint (see Figure 11b–d). The edge ISZ of the PBHT joint is inherited from the as-prepared ISZ formed at 1230 °C, whereas the central ISZ of the PBHT-processed joint is formed at 1150 °C. Consequently, the initial liquid phase compositions are different during two isothermal solidification processes. Therefore, both regions exhibited different image contrasts.

### 3.3. Creep Properties and Fracture Mechanism of Brazed Joints

The high-temperature creep properties of the as-prepared and PBHT-processed joints are tested at 980 °C/100 MPa five times, respectively. The test results show that the average creep life of as-prepared joints is 188 h, and that of PBHT-processed joints is 243 h. It can be found that the creep life of the PBHT-processed joint is 23% higher than the as-prepared joint. The improved creep life can be ascribed to the removal of most of the low-melting-point phases after PBHT and decrease in the content of brittle borides, improving the crack resistance of the weak point of the joint during the high-temperature creep process. Additionally, the elemental interdiffusion is enhanced after PBHT; γ′-forming elements are transferred into the bonding area and increase the volume fraction of the precipitation strengthening phases, namely the γ′ phase, which is also beneficial to the high-temperature creep properties.

**Figure 12:**
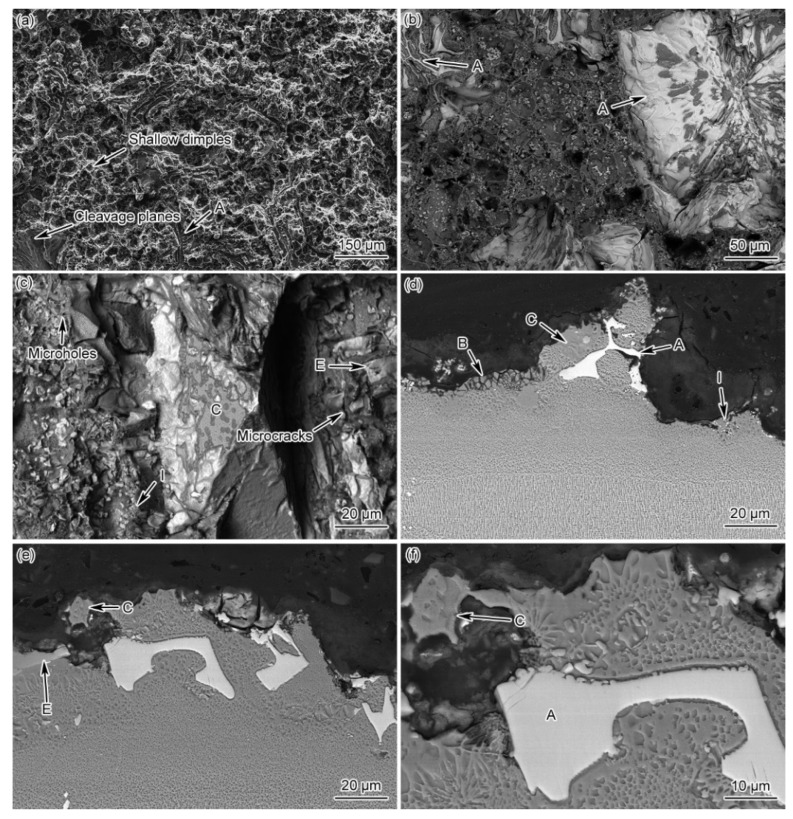
The morphologies of the fractured surface and longitudinal section of the as-prepared joints (A, B, C, E, I represent Area-A-B-C-E, and -I, respectively): (**a**–**c**) fracture morphology and (**d**–**f**) longitudinal section morphologies.

Figure 12 presents the fractured and longitudinal morphologies of the as-prepared joint. There are certain amounts of micro-holes and microcracks on the fracture surface, and the fracture characteristics belong to a quasi-cleavage fracture with a river-like pattern. The fracture mode of the as-prepared joint is determined as a cleavage-dominant mixed fracture. In addition, the fracture surface is covered with many white-colored compound particles (Letter I in Figure 12c), which do not exist in the original as-prepared joint. The EDS results of the fracture surface are presented in Table 3, showing that the newly formed particles contain a high amount of Cr, which can be inferred as a boride transferred from the M_3_B_2_ phase.

Moreover, phase I, phase A, γ + γ′ eutectic, phase C, and phase E are also observed on the fracture surface and longitudinal section (Figure 12b–f). Therefore, it can be inferred that, due to the enrichment of B in the joints, net-like distributed boride particles (phase I) precipitated at the grain boundary during the creep test under the influence of high temperature and stress, producing the undesirable microcracks. The boride network enables these microcracks to rapidly connect and propagate along grain boundaries. In the meantime, microcracks are also initiated at the phase boundaries between the matrix and other compound phases in the ASZ of the joint. These interphase cracks are associated with the grain boundary cracks and expeditiously propagated, leading to the joint fracture. In short, the high content of brittle compounds and low-melting-point eutectics in the as-prepared joint led to joint failure.

**Figure 13:**
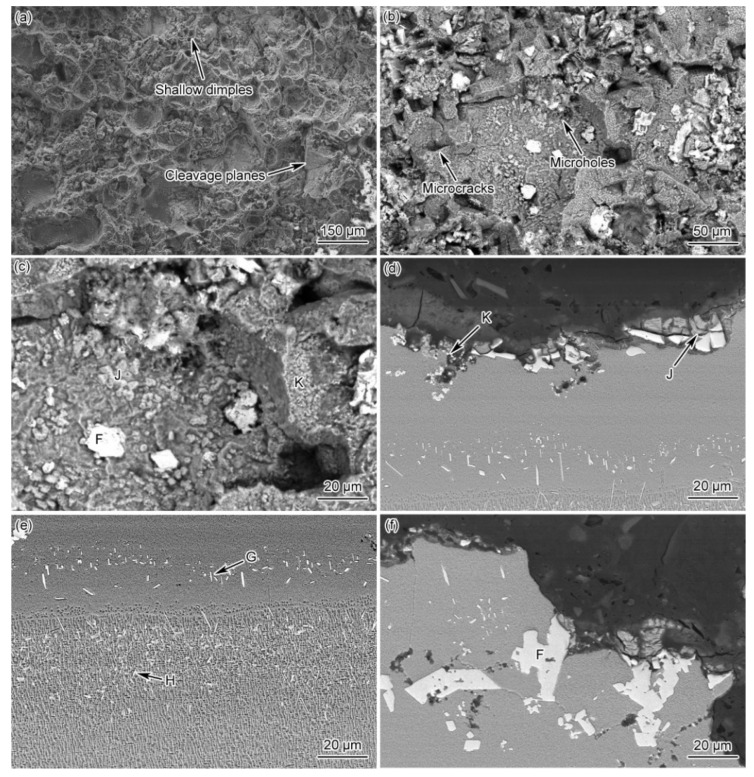
The morphologies of the fractured surface and longitudinal section of the PBHT-processed joints (F, G, J, K represent Area-F-G-J, and -K, respectively): (**a**–**c**) fracture morphology and (**d**–**f**) longitudinal section morphologies.

Figure 13 presents the fracture morphology of the PBHT-processed joint, showing a large number of shallow dimples and a few cleavage planes on the fracture surface. Therefore, the fracture mode of the PBHT joint can also be considered as a hybrid ductile and quasi-cleavage fracture, which is dominated by ductile fracture. It is observed that there are a large number of slightly fragmented blocks and two white-colored compounds with different sizes and shapes on the fracture surface (Figure 13b,c). The EDS results (Table 3) reveal that the composition of the compound group with a larger particle size (phase J in Figure 13d) is similar to the slightly fragmented block-shaped phase F, whereas the compound with a finer particle size (phase K in Figure 12d,f and Figure 13c) is rich in Cr. The above phenomena can be analyzed as follows: the microcracks are initiated and propagated from the block-shaped phase F in the ASZ during the creep process; phase F is decomposed and fragmented under the effect of high temperature and stress; and part of the fragmented phase F is evolved into the large-size boride particles (phase J). During continuous degradation and decomposition of phase F, a large number of B and boride forming elements are released and reprecipitated at grain boundaries, gradually forming fine boride particles (phase K). Under the action of creep stress, the cracks are initiated from phase F, phase J, and phase K, and propagated along the grain boundaries, leading to the fracture of the joint. Additionally, there are no cracks or micro-holes in the DAZ and the edge ISZ (Figure 13e), indicating that phase G and phase H do not render any adverse effect on the high-temperature creep performance of the PBHT joints.

By comparing the fracture morphology and creep life of the as-prepared and PBHT-processed joint, it can be found that the elimination of Area-A, γ + γ′ eutectic, Area-C, Area-D, and Area-E from the ASZ of PBHT-processed joined reduced the potential sources of creep cracks. The expansion of ISZ and reduction of ASZ improved the ability of coordinated deformation of the joint under the action of stress [34]. All these factors played a positive role in improving the creep resistance of the PBHT-processed joints. Meanwhile, the volume fraction and uniformity of γ′ precipitates in the PBHT-processed joint have been increased, inhibiting the movement of dislocations and formation of creep holes during the creep process, which is also beneficial to the creep performance [38]. Therefore, it is recommended to employ a high-temperature PBHT to remove the detrimental phases and improve the microstructural uniformity of the joints. Thus, a better brazed joint is expected.

## 4. Conclusions

In summary, the influence of PBHT on microstructure and creep behavior of brazing joints of a nickel-based single crystal superalloy is systematically investigated. The following conclusions can be drawn from the current study:

1. The microstructure of the as-prepared joint, brazed at 1230 °C for 30 min, consisted of ISZ and ASZ. In the ISZ, the size and volume fraction of γ′ precipitates increased with the decreased of distance from the BM. The ASZ was mainly composed of γ matrix, skeleton-like M_3_B_2_ phase, γ + γ′ eutectic, reticular G phase, block-shaped M_3_B_2_, block-like M_3_B_2,_ and precipitated γ′ phase. The size of the γ′ precipitates close to the skeleton-like M_3_B_2_ phase was obviously higher than the γ′ precipitates near the reticular G phase.
2. After PBHT, the joint was composed of γ matrix and block-shaped M_3_B_2_ phase, and the low-melting-point eutectics were removed. The central ISZ was formed by the secondary isothermal solidification of the re-dissolved ASZ of the as-prepared joint. The edge ISZ, which contained lots of lath-like M_3_B_2_, was inherited from the original ISZ of the as-prepared joint. The interdiffusion of elements resulted in a fuzzy interface between the central ISZ and edge ISZ. Moreover, the size of γ′ precipitates in both ISZs was extremely finer than that in the as-prepared joint. The newly formed DAZ was located on the BM adjacent to the edge ISZ, where numerous elliptic flake-like M_3_B_2_ precipitates gradually sparsed with the increase of distance from the ISZ/DAZ interface.
3. The creep life of the as-prepared joint can be improved by 23% after PBHT. During the creep process, the cracks of as-prepared joint were mainly initiated and propagated along the γ + γ′ eutectic, as well as the skeleton-like M_3_B_2_ phase and newly-formed net-like distributed boride particles at grain boundaries, showing a mixed fracture mode, i.e., ductile and cleavage fracture. In the case of the PBHT-processed joint, the cracks were mainly nucleated from the M_3_B_2_ phase and fine boride particles, resulting in a ductile-dominant mixed fracture.

## Figures and Tables

**Table 1:** The chemical composition of the base material and filler (at.%).

Material	Cr	Co	Mo	W	Re	Al	Ti	Ta	Hf	C	Si	B	Ni
Superalloy	3.3–4.5	12–15	0.5–0.75	2–2.6	1–2	12–15	-	2–3	0.02–0.05	0.05–0.15	-	-	Bal.
Filler metal	12–15	9–11	1–2	1–2	-	2–4	1–2	-	-	-	5–8	6–9	Bal.

**Table 2:** EPMA analysis of different regions of the as-prepared and PBHT-processed joints (at.%).

	ISZ	Area A	Area B	Area C	Area D	Dark Area of Area E	Bright Area of Area E	γ Matrix Around Area A	γ Matrix Around Area C	Area F	Area G	Needle-like Phase H	Flaky Phase H
Joint state	As-bonded	As-bonded	As-bonded	As-bonded	As-bonded	As-bonded	As-bonded	As-bonded	As-bonded	Heat treated	Heat treated	Heat treated	Heat treated
B	0.00	39.45	0.00	13.12	38.81	40.55	40.62	0.00	0.00	35.23	30.47	19.11	14.58
Al	8.61	0.63	11.21	0.92	0.12	0.11	0.11	9.00	0.45	0.40	1.21	3.71	3.12
Si	2.47	0.00	5.85	1.21	0.70	0.22	0.65	3.51	13.84	0.14	0.46	1.15	1.25
Ti	0.57	0.64	1.57	3.54	0.43	0.48	0.65	0.64	0.16	0.91	0.62	0.39	0.59
Cr	9.46	18.91	3.54	5.39	38.53	31.68	19.04	8.68	2.24	7.15	7.12	3.54	3.88
Co	12.85	4.19	8.4	18.05	5.71	4.38	4.02	12.20	9.16	9.54	10.15	12.51	12.07
Ni	61.56	10.61	66.86	54.44	6.45	6.53	7.80	62.69	73.86	15.32	28.29	47.78	51.14
Mo	0.78	7.58	0.14	0.13	3.85	6.54	8.71	0.46	0.05	6.85	3.80	1.21	1.51
Hf	0.00	0.05	0.00	0.25	0.00	0.04	0.00	0.00	0.00	0.00	0.00	0.00	0.00
Ta	0.68	2.47	1.86	2.72	0.87	1.46	2.82	0.79	0.10	5.89	2.84	3.28	4.17
W	2.27	13.49	0.51	0.23	1.98	4.91	13.46	1.48	0.14	16.20	13.59	5.25	5.82
Re	0.75	1.98	0.06	0.00	2.55	3.10	2.12	0.55	0.00	2.37	1.45	2.07	1.87
Total	100	100	100	100	100	100	100	100	0.00	100	100	100	100

**Table 3:** EDS analysis of different phases at the fractured surfaces of the as-prepared and PBHT-processed joints.

at.%	Phase I in the As-Bonded Joint	Phase F in the PBHT Joint	Phase J in the PBHT Joint	Phase K in the PBHT Joint
O	15.32	38.51	37.29	53.22
Al	7.17	1.56	2.02	0.00
Ti	0.00	1.17	1.24	0.74
Cr	32.4	10.14	21.12	26.08
Co	11.67	6.93	4.90	2.71
Ni	33.44	24.10	16.95	9.28
Mo	0.00	1.14	3.14	3.07
Ta	0.00	2.35	2.05	0.00
W	0.00	14.10	11.29	4.90
Total	100	100	100	100

## Data Availability

The raw data required to reproduce these findings cannot be shared at this time as the data also forms part of an ongoing study.
